# A Comparative Analysis of Student and Practising Nurses’ Health Literacy Knowledge in Ghana

**DOI:** 10.3390/healthcare9010038

**Published:** 2021-01-04

**Authors:** Adwoa Owusuaa Koduah, Padmore Adusei Amoah, Jacob Oppong Nkansah, Angela Y. M. Leung

**Affiliations:** 1 Center of Gerontological Nursing, School of Nursing, The Hong Kong Polytechnic University, Hong Kong SAR, China; owusuaa.koduah@connect.polyu.hk; 2 Institute of Policy Studies, Asia Pacific Institute of Ageing Studies, and School of Graduate Studies, Lingnan University, Hong Kong SAR, China; 3 School of Graduate Studies, Lingnan University, Hong Kong SAR, China; oppongnkansah@ln.hk; 4 Centre for Gerontological Nursing, WHO Collaborating Centre for Community Health Services (WHOCC), School of Nursing, The Hong Kong Polytechnic University, Hong Kong SAR, China; angela.ym.leung@polyu.edu.hk

**Keywords:** health literacy, health literacy knowledge, nurses, student nurses, practising nurses, Ghana

## Abstract

This study examined student and practising nurses’ health literacy knowledge, and its correlates in Ghana. It was underpinned by an adapted version of the Institute of Medicine’s (IOM) conceptual framework of health literacy. We used convenience and snowball sampling techniques to collect data from 876 nurses (477 student nurses and 399 practising nurses) in a cross-sectional survey from February 2019 to June 2019. The respondents were drawn from all the former ten administrative regions of Ghana. Approximately 75.4% of the respondents had heard of health literacy. However, health literacy knowledge was generally low (average score of 6.6 out of 20) among both groups, with student nurses (average score of 5.8 out of 20) having significantly lower scores than practising nurses (average score of 7.4 out of 20). Factors associated with health literacy knowledge among student nurses included gender (male, B = −0.499, *p* < 0.01), trust in others (B = −0.874, *p* < 0.001), cultural values (B = 0.276, *p* < 0.001), year of study (B = 0.244, *p* < 0.05), and frequency of curative care use (B = −0.236, *p* < 0.05). For practising nurses, trust (B = −1.252, *p* < 0.01), cultural values (B = 0.357, *p* < 0.01), and working experience (B = 0.612, *p* < 0.01) were associated with their health literacy knowledge. Thus, responses targeted at gaps in health literacy knowledge of student and practising nurses must be sensitive to personal characteristics (e.g., gender), social values (e.g., issues of trust, and cultural beliefs and practices), as well as factors relating to nursing education and experience.

## 1. Introduction

Health systems globally require the public and patients to participate in health service delivery [1,2]. Accordingly, the public must be knowledgeable or sufficiently educated on the fundamental and, where applicable, intricate aspects of health service delivery, including how the health system operates to ensure efficiency and, ultimately, desirable health outcomes. To achieve this objective, health professionals, particularly nurses, must play a decisive part because their role is typically at the interface between the public and patients needing healthcare. They are the largest patient education providers and are best placed to transmit the requisite health information [2,3,4,5] to the public and their patients. For instance, in Ghana, 58 per cent of the 115,650 public sector health workers are nurses, which shows their importance to caregiving [6]. However, for nurses to execute this task, they must be well-trained themselves [7,8]. Nurses must be able to assess deficiencies in patient health literacy and prepare them to participate in their healthcare programmes [4,9].

Health literacy has become an essential asset in modern health promotion strategies [10,11]. It is ‘linked to literacy and entails people’s knowledge, motivation and competences to access, understand, appraise, and apply health information in order to make judgments and take decisions in everyday life concerning healthcare, disease prevention and health promotion, to maintain or improve quality of life during the life course’ [12]. 

People with sufficient health literacy demonstrate sound judgement about matters that affect their health, and they tend to avoid deleterious health behaviours, such as substance abuse and the drawbacks associated with leading sedentary lifestyles [12,13]. Moreover, having sufficient health literacy is associated with competence in engaging with the health system, including taking advantage of policies to promote healthcare access and adopting preventive health measures [14,15]. Consequently, sufficient health literacy is associated with better health outcomes—making it an essential health promotion component [13,16].

Unfortunately, extant evidence suggests that many nurses have low health literacy, while others are unaware of the concept and its application and relevance to practice [9,17]. Many nurses have limited skills in identifying population groups at risk of low health literacy, while others have inadequate experience in screening and assessing patients’ health literacy [2,4,17]. What is more, there are indications that health literacy knowledge of practising and student nurses may differ [9,18]. Student nurses refer to people enrolled in various health science training schools (typically at degree or sub-degree levels) to become professional nurses in a specific or general nursing area [8,17,19]. Practising nurses encapsulate graduates from various nursing training institutions who have obtained the relevant license to practice nursing [20]. Practising nurses usually have different roles and specialities depending on their training and place of work [20]. Working experience and regular interactions with other health professionals, which tend to be common among practising nurses, are associated with health literacy [18]. This signifies a likelihood of low health literacy knowledge among student nurses [5,17,19]. Despite these potential differences in nursing experience and training, existing research on health literacy knowledge among nurses has dwelled extensively on either student nurses [5,8,17] or practising nurses [2,18,21,22]. No study has examined the health literacy knowledge of the two types of nurses concurrently, leaving a significant gap in the existing literature.

For student nurses, health literacy knowledge has been associated with the nursing education curricula [9,23,24]. Among practising nurses, the conditions of their working environment are considered an important determinant of health literacy knowledge [2,22]. However, most evidence on nurses’ health literacy knowledge originates from advanced economies due to little research in regions such as sub-Saharan Africa and other low-income settings [25,26]. In places such as Ghana, existing health literacy studies have focused on street children and youth [27,28]; undergraduate students [29]; maternal health [30]; geographical perspectives [31]; general populations [32]; and cultural aspects of health literacy among nurses [21]. All this notwithstanding, a consensus from these studies provides clear evidence of low health literacy and low health literacy knowledge among all these groups. 

### 1.1. Aims of This Study

**Figure 1:**
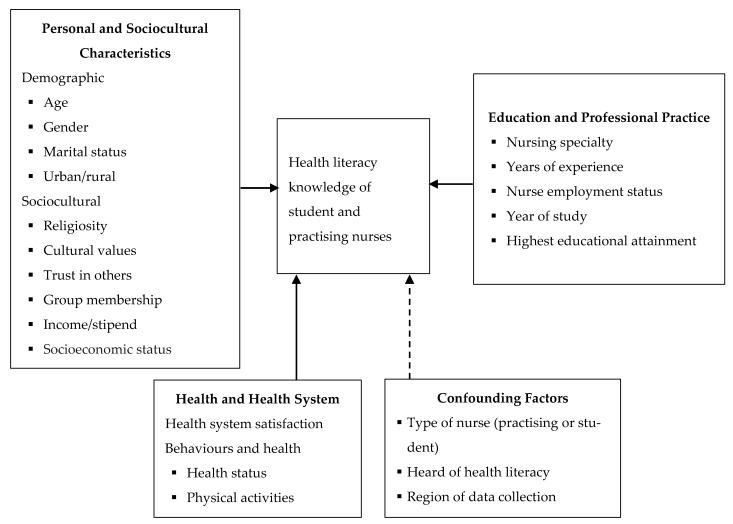
Conceptual framework of the correlates of health literacy knowledge of student nurses and practising nurses. **Source:** Adapted from the Conceptual Framework for Health Literacy [10].

This study aims to examine the health literacy knowledge of student and practising nurses in Ghana and identify the factors associated with that knowledge. These factors are identified through an adapted version of the Institute of Medicine’s (IOM) conceptual framework of health literacy [10] (see Figure 1). By comparatively exploring the factors associated with the health literacy knowledge of student and practising nurses, this study offers important insights into specific ways of intervening in nursing education and practice to promote health literacy principles in health service delivery. The study will also represent a significant shift in existing research by examining not only the state of health literacy knowledge of the two groups of nurses but also the factors associated with it. 

### 1.2. What Accounts for Health Literacy and Health Literacy Knowledge? Conceptual Perspectives

In both low-and-high-income countries, limited health literacy knowledge among health professionals is often associated with a multiplicity of factors relating to the health education systems, working conditions and equipment, personal characteristics (including age, gender, working experience) and the effects of prevailing socio-cultural norms and practices. These factors are aptly presented by IOM’s conceptual framework for health literacy. The framework identifies three domains that are responsible for health literacy and its subsequent influence on health outcomes: firstly, culture and society (values, identity, preferences and behaviours accepted in a given society); secondly, the health system itself; and thirdly, the education system [10]. Specific elements under these three domains help gauge the factors that can contribute to the knowledge and experience that individuals have regarding health literacy. Indeed, as the IOM argues, the development of appropriate procedures, policies, and programmes to improve the public’s health literacy requires personnel who have a clear understanding of the problem of health literacy from multi-sectoral perspectives [10]. 

We adapted these domains to make them relevant to the aims of this study. The revised domains include *personal and socio-cultural characteristics; health and health system; and education and professional practice (*Figure 1*).* The domain relating to culture, society, and personal characteristics encapsulates factors associated with cultural and societal precepts, such as reliance on cultural principles, religiosity, trust, and group involvement. This domain includes personal characteristics such as age, gender, income, marital status, and residence location (e.g., rural or urban). We combine cultural and demographic factors because in many societies in developing countries, personal characteristics and their roles in societies are shaped by the prevailing cultural practices and norms [33].

The health and health system domain encompasses policies and structures designed to promote health, prevent diseases and offer curative services [10]. The characteristics of a given health system in terms of the logistics, equipment, personnel and the type of health facilities can affect health literacy and health literacy knowledge and people’s experience [2,10]. We conceptualise this domain to include the health status and health behaviours of the nurses and their overall satisfaction with the health system. The health personnel’s health-related characteristics will help gauge health literacy itself, while their satisfaction with the health system provides insights into their health literacy experiences.

Finally, the education and professional practice domain emphasise the fact that academic training and professional experience are likely to affect health literacy knowledge of nurses [21]. To consider the situation of both students and practising nurses, this domain comprises factors such as years of working experience, the year of study, and nursing speciality. The domain brings to fore the fundamental role of literacy and experience in health literacy and health literacy knowledge, as well as the application of the information/knowledge that people obtain [10,12]. Thus, together, the three domains of the health literacy framework provide a holistic view of the potential correlates of health literacy knowledge of nurses.

## 2. Methods

### 2.1. Study Design

Data for this study were derived from a cross-sectional survey that was conducted in healthcare facilities and nursing training institutions across Ghana. The survey took place from February 2019 to June 2019 in all the ten administrative regions of Ghana as part of a broader social epidemiological study. The Council for Scientific and Industrial Research (CSIR) of Ghana approved the study protocol (RPN 005/CSIR-IRB/2018).

### 2.2. Sampling

We used convenience and snowball sampling techniques to select respondents in nursing training schools (irrespective of their speciality and level of education/training being pursued) and practising nurses from all kinds of health facilities. Student nurses had to be in their second year of study, while practising nurses were included in the survey if they had at least two years’ working experience. This was to ensure that both groups had had adequate opportunity to study or obtain some health literacy experience. Many practising nurses in Ghana often return to school for advanced education either privately or with support from the Ministry of Health (for those in the public sector) within the first five years of practice [6]. Therefore, nurses who were pursuing either part-time or full-time further education after more than two years of practice were considered as practising nurses in this study, given their significant clinical experience. This approach ensured that a clear distinction could be drawn between student and practising nurses during the recruitment of respondents. Given that respondents were mostly literate, all the questionnaires were self-administered, via either online (using the Qualtrics ^XM^ platform (Qualtrics LLC., Washington, DC, USA)) or paper-based questionnaire. Either way, trained interviewers were on hand to support respondents who had challenges in completing the survey. The goal was to ensure a representative sample by recruiting at least 384 respondents each for the student and the practising nurses [34]. This minimum sample size was derived using the formula: *Ns =* (*Np*)(*p*)(1 − *p*)/(*Np* − 1)(*B* / *C*)^2^ +(*p*)(1 − *p*), where *Ns* = total sample size needed; *Np* = size of population; *p* = proportion expected to answer a certain way; B = acceptable level of sampling error; C = Z statistic associate with confidence interval. These criteria were considered: 95% confidence level; sample error of 0.05; and assumption that 50% of respondents would adequately respond to questions [34].

### 2.3. Measures

#### 2.3.1. Dependent Variable

*Health literacy knowledge:* We measured this using an adapted version of Part I of the Health Literacy Knowledge and Experience Survey Instrument (HLKES) [17,24]. The original instrument consists of 29 items which measures knowledge of health literacy in five content areas, namely “basic facts of health literacy (6 questions); health literacy screening (6 questions); consequences associated with low health literacy (4 questions); guidelines for written and healthcare materials (11 questions); and evaluation of health literacy interventions (2 questions),” [8]. The instrument questioned respondents in all these content areas by asking them to answer questions that demonstrated their familiarity with and knowledge of health literacy. Respondents had to select the right answer (among four options). We used only the Part I of the instrument to reduce the bias against the student nurses sample who were less likely to have gained adequate clinical experience on health literacy as observed elsewhere [9]. To make the original instrument applicable to the study context, nine of the items were removed through face validity assessment by the research team and an independent health literacy researcher. Some of the questions removed includd those that were most difficult and unfamiliar to many nurses, including those in high-income countries [2].

Items that were removed included those relating to specific health literacy screening and measurement tools/techniques, as well as those that were more relevant to Western contexts where the instrument was developed. For example, the following questions were removed: Which of the following statements best describes the Fry Method? Which statement best describes the Test of Functional Health Literacy (TOFHL)? “The Rapid Estimate of Adult Literacy in Medicine (REALM) is an instrument used to…” and “Low health literacy levels are more common among…?” These questions were less likely to be answered correctly or understood by respondents given the novelty of health literacy in public health and nursing education in African settings, and Ghana in particular [25]. This meant that the respondents’ correct scores could range from 0 to 20 compared with the original instrument, which scores from 0 to 29. Overall, the new instrument we used had adequate reliability with a Cronbach’s alpha of 0.71 in our study. In the analyses, all correct and incorrect responses were scored as ‘1’ and ‘0’ respectively. All scores were summed to provide a total score of health literacy knowledge.

#### 2.3.2. Independent Variables

*Personal and socio-cultural characteristics:* We measured several variables under this domain including age (in years), gender (male, female), marital status (married, divorced, widowed, separated, living together as married, or single), area of residence (urban/rural), religiosity (“Religion is very important in my life”, with response options on a five-point Likert scale: strongly disagree to strongly agree), cultural values (“I have a deep respect for traditions preservation of customs and beliefs”, with response options on a five-point Likert scale: strongly disagree to strongly agree), trust in others (yes/no), membership of an association (yes/no); monthly income/allowance; and self-perceived socioeconomic status (SES, rated from 1 (low) to 10 (high)).

*Education and practice characteristics:* We measured these variables: nursing speciality; years of experience (for practising nurses); year of study (for students); and highest educational attainment as detailed in Table 1.

*Health and health system characteristics.* This domain measured respondents’ satisfaction with the health system (seven-point Likert scale from completely dissatisfied to completely satisfied, with a high score indicating more satisfaction and vice versa); self-rated health status (five-point Likert scale from poor to excellent); engaging in physical activities (five-point Likert scale from never to daily); use of curative healthcare (the frequency with which the nurses visited the emergency room because of ill health in the past 12 months; measured on five-point Likert scale from never to daily).

*Covariates*. We controlled for the following variables: whether the respondent had heard of health literacy (yes/no); the region of data collection; type of nurse (student/practising nurse).

### 2.4. Data Analyses

At the outset, our analyses comprised of descriptive statistics to provide an overview of the respondents’ characteristics and status as regards the key variables, using frequencies and percentages for categorical variables and mean (with standard deviation, SD) for continuous and ordinal variables. We subsequently carried out a Spearman’s correlation analyses to identify initial variables associated with health literacy knowledge as the data for both student (Shapiro–Wilk test, *p* = 0.001) and practising (Shapiro–Wilk test, *p* = 0.001) nurses were not normally distributed. The final set of analyses involved an ordinal logistic regression. We used the total score of health literacy knowledge as the dependent variable. We included all variables showing a significant relationship with health literacy knowledge in the correlation analyses as independent variables. The analysis was conducted with SPSS version 26 (SPSS Inc., Chicago, IL, USA). Significant associations were evaluated at *p* < 0.05.

## 3. Results

We received 1039 responses (comprising 270 paper-based and 769 online responses). However, the analyses presented in this study were based on 250 paper-based and 626 online responses after removing significantly incomplete cases. Table 1 shows descriptive statistics of all the variables in the study. Most respondents were females (56.5%). The respondents emerged from each of the ten administrative regions of Ghana at the time of the survey. Their educational levels spanned from Certificate in Nursing to Masters’ degrees, specialising in different nursing sub-fields (see Table 1). For most respondents, religion and cultural values were a significant part of their lives. Furthermore, most (65.6%) of them felt that they could not trust other people in their communities completely. The majority (65.5%) of them were members of at least one association. The practising nurses had an average work experience of almost five years, while the students were mostly in their second (46.4%) and third (48.1%) years of study. On their health-related issues, most respondents rated their health in favourable terms. Only about 33.4% of respondents were satisfied with the health system. Approximately 75.4% of them had heard of health literacy, which was significantly higher among student nurses. However, their knowledge of the health literacy as measured by the HLKES instrument showed a generally low awareness among both groups (average score of 6.6 out of 20), although practising nurses (average score of 7.4 out 20) were significantly more knowledgeable (albeit low) than student (average score of 5.8 out of 20) nurses. The two groups also differed in age, gender, educational attainment, income, marital status, trust, socioeconomic status, physical activity, use of curative services and awareness of health literacy. Table 1 shows the descriptive results of all variables we measured.

Student nurses were less likely to have sufficient knowledge of health literacy compared to practising nurses. Among the student nurses, sex (being male, B = −0.499, *p* < 0.01), frequency of curative care use (B = −0.236, *p* < 0.05), and having trust in others (B = −0.874, *p* < 0.001) were negatively associated with health literacy knowledge. However, cultural values (B = 0.276, *p* < 0.001) and year of study (B = 0.244, *p* < 0.05) showed positive associations with health literacy knowledge. For practising nurses, factors associated with their health literacy knowledge included trust in others (B = −1.252, *p* < 0.01), cultural values (B = 0.357, *p* < 0.01), and working experience (B= 0.612, *p* < 0.01). In the overall sample, those married (B = −1.930, *p* < 0.05), and living together with someone as married (B = −1.357, *p* < 0.05) were less likely to have health literacy knowledge than respondents who identified themselves as single. Income/stipend (B = 0.001, *p* < 0.001) and cultural values (B = 0.378, *p* < 0.01) were positively associated with health literacy knowledge. However, SES (B = −0.221, *p* < 0.01) and trust in others (B = −0.939, *p* < 0.001) were negatively associated with health literacy knowledge. These results are presented in Table 2.

## 4. Discussion

This study examined the health literacy knowledge and factors associated with that knowledge among students and practising nurses in Ghana. The health literacy knowledge of both student and practising nurses was low. Although there were differences in the factors associated with the health literacy knowledge of the student and practising nurses (e.g., sex and frequency of curative health care), there were also similarities in the factors associated with the health literacy knowledge of both groups (e.g., cultural values).

While most of the nurses had heard of health literacy, we found that health literacy knowledge was low. This disparity shows that health literacy knowledge goes beyond mere familiarity with the concept. Health professionals must be deliberately trained to become knowledgeable in health literacy for personal and professional use. This finding is consistent with previous research [2,9,17], and it lends support to assertions that many health professionals in Ghana have significant gaps in their health literacy knowledge [21].

In many instances, low health literacy knowledge among nursing students is associated with low exposure to the concept (through practice), and lack of relevant training and practical experience [5,9]. Some argue that the current young nurses in Ghana are relatively inexperienced because there are too few clinical mentors to guide them [6]. In the absence of adequate comprehensive practical experience and adequate mentors, it is understandable why student nurses have low health literacy knowledge [5,9]. Correspondingly, nursing training schools must create more opportunities for students to learn about health literacy through clinical experience.

However, the above suggestion raises questions about why practising nurses in this study also had low health literacy knowledge. The situation compels a more in-depth examination. As argued in the existing literature, there are critical gaps in the nursing education curriculum in Ghana and many other places pertaining to health literacy, which explains the low awareness of the concept [2,18,21]. Given such gaps, nursing practice may only add experiential knowledge instead of theoretically grounded skills in health literacy, such as identifying populations at risk of low health literacy and how to communicate vital information to patients effectively. Our findings imply a need to rethink the current curricula of nursing education to include adequate content on health literacy. For current practising nurses, workshops on health literacy and its application in clinical settings are non-negotiable. Without such opportunities for training, nurses are likely to rely on untested approaches in their practice (e.g., recommending unapproved spiritual care to patients), which can be injurious to patient outcomes [see 21]. However, provision of opportunities for student and practising nurses to learn about health literacy must be made alongside the creation of favourable conditions for nurses to apply their knowledge. For instance, appropriate tools (e.g., health literacy screening tools, and other educational materials) must be readily available to nurses as their absence can render their knowledge useless to care [18,21,26].

Among the student nurses, being male and use of curative health services were negatively associated with health literacy knowledge. The finding on gender is inconsistent with that of a population-based study in Ghana, which found that males have greater health literacy and related knowledge than females [36]. That study attributed high health literacy among males to their dominance in education as well as the prevailing patriarchal norms, which inadvertently incite males to claim positive health outcomes and health-related efficacies to keep up with social expectations. This study contributes to another perspective because health literacy knowledge was measured using an objective instead of a subjective instrument. This implies that even if males were to have better health literacy knowledge than females in the general population, females are likely to be better with the right training and support. Indeed, a related study of health literacy among undergraduate students in Ghana found no difference between males and females [29]. Future studies can explore why and how female nursing students are likely to have greater health literacy knowledge than their male counterparts, to provide a platform for appropriate nursing education and development.

The inverse relationship between utilising curative services and health literacy knowledge among the student nurses supports the widely held position that having sufficient health literacy (and knowledge of it) promotes positive health outcomes [1,12]. With better health literacy knowledge, people are less likely to utilise curative health services. Such individuals patronise preventive health services to stay healthy [10,12]. This study adds that such knowledge of health literacy is relevant not only to the public but also to health professionals.

Aside from gender and the use of curative services, which were solely associated with the health literacy knowledge of nursing students, all the other significant correlates of health literacy knowledge were common to both student and practising nurses. Principally, the common correlates included trust in others, cultural values, and year of study/years of experience. These similarities give credence to the tenets of the conceptual framework of health literacy. This is because the factors identified correspond to two domains of the framework namely, culture, society and personal characteristics (trust and cultural values), and, secondly, education and professional practice (education and work/work experience) [10]. Trusting relations with others negatively predicted the health literacy knowledge of both groups of nurses. This finding resonates with the social network and health theory, which suggests a strong connection between health, health behaviours, health-related efficacies and social networks [32,37,38]. Trusting relations afford people opportunities to learn and seek support from others in their communities. In professional settings, trust promotes collaborative working environments and effective patient-provider communication [10,39,40]. Inevitably, such environments can provide opportunities for improving the health literacy and related knowledge of both patients and health professionals. However, this study shows that the importance of trust in health literacy knowledge depends on the calibre of people in a given social network. Thus, the negative association between trust and health literacy knowledge raises a question of the state of the health literacy knowledge of health professionals and the public in general. This study underscores previous ones that suggest low health literacy knowledge among nurses [21] and low health literacy among the public [14,36]. Thus, low health literacy knowledge in both clinical and non-clinical communities partly explain why nurses in this study had low health literacy knowledge as they likely associated with, and trusted people who probably had low health literacy knowledge.

Another finding from this study corroborates the above arguments; nurses who were married or living together with their partners had lower health literacy knowledge than those who were single. People in intimate or close social relationships are likely to trust their partners for health information, making them vulnerable to miseducation if the people in their networks or themselves have low health literacy knowledge [41]. Besides, the responsibilities associated with being in committed relationships can have detrimental effects on health-related knowledge and choices [42]. These arguments are contrary to some existing studies which posit that being in committed relationships can create opportunities to learn more about health-related issues [43]. Given the importance of social networks and trust, strategies to improve health literacy knowledge among nurses must be attentive to their social networks in clinical and non-clinical communities as other people can influence the nurses’ ability to apply their knowledge.

We also found a positive association between cultural and traditional values and health literacy knowledge among both student and practising nurses. According to the IOM health literacy framework, cultural precepts and practices are fundamental to health literacy and knowledge, as people often interpret health and healing matters from cultural perspectives [10]. Hence, it would be unrealistic to detach nurses from their socio-cultural environment since many cultural beliefs and practices are consciously and unconsciously ingrained in nursing practices in Ghana and similar places [21]. If anything at all, our finding indicates that cultural values and orthodox knowledge can complement each other if both are well-understood and applied. A nursing education curriculum and training focusing on health literacy must target cultural competency so that nurses can elicit useful aspects of local cultures in care delivery and patient education in ways that do not compromise quality of care. In the absence of such cultural considerations, health literacy promotion strategies are unlikely to achieve their goals. The IOM contends that “there is a need to understand the independent contributions of cultural competence and health literacy to patient safety, as well as the interactions between cultural competence and health literacy”, [10]. In view of our findings, the IOM’s assertion should not be limited to the public but should be extended to nursing training.

Moreover, the experience of nurses, in terms of years of nursing education and nursing practice, was associated with health literacy knowledge. This implies that deliberate efforts to train and expose nurses to health literacy through health education and practice can improve their health literacy knowledge. Existing studies show that nursing students’ overall health-related knowledge increases as they move along their study programmes [19]. A similar explanation can be offered for practising nurses. Extended years of practice can unconsciously transmit health literacy skills to nurses. In a study of nurse practitioners’ health literacy knowledge in the US, Cafiero [2] found that nurses whose work focused on speciality practices had higher health literacy experiences than those in episodic care settings. Nurses who had intensive and repetitive work experience were more likely to have better health literacy knowledge [2]. Concerning our study area, these findings assure that if health facilities and education institutions are furnished with health literacy resources, nurses and other health professionals will undoubtedly gain more opportunities to improve and apply their health literacy knowledge [21].

We also found that income/stipend was positively associated with health literacy knowledge, but SES was negatively associated with health literacy knowledge. These findings partially explain the conceptual complexity of SES [44]. On the one hand, while income is considered a part of SES, other aspects such as social networks and social support can be deleterious to nurses’ health literacy knowledge if the people in the network have low health literacy regardless of their position in the society [41]. On the other hand, having adequate income creates avenues for people to improve health-related knowledge through educational opportunities, and increases the ability to consult appropriate sources for health information [45]. Among nurses, such financial resources can increase their chances of consulting other health professionals instead of relying on personal resources alone. Such practice is known to improve health literacy knowledge [18].

### Limitations of the Study

The study did not deal with the state of specific domains of health literacy knowledge, such as basic facts of health literacy, and health literacy screening. Several other studies have addressed such research gap, and they appear to have consistent findings [2,9,18]. In addition, as we removed some of the items from the original instrument, it would have been problematic to present the characteristics of the various domains of health literacy knowledge. Moreover, while there were some socio-demographic differences between the practising nurses and student nurses, our discussion has not explored these differences in detail as that was not the primary focus of the study. Future studies can address these issues using representative data because our data were based on convenience sampling.

## 5. Conclusions

We found evidence that factors relating to all three aspects of the adapted health literacy framework were associated with the nurses’ health literacy knowledge. Being male and using curative health services was uniquely associated with student nurses’ health literacy knowledge. However, trusting relations with others, cultural values and education/work experience were commonly associated with student and practising nurses. These findings demonstrate the consistency, but also the variations in the underlying factors of the health literacy knowledge of student and practising nurses. The findings offer significant insights into potential areas of emphasis as part of measures aiming to incorporate health literacy in nursing education and address gaps in health literacy knowledge of practising nurses. For instance, efforts to create opportunities for student and practising nurses to acquire health literacy knowledge in their studies and professional practice respectively can incorporate personal characteristics (e.g., gender), social values (e.g., health literacy of their trusting acquaintances, and cultural beliefs and practices), and their nursing education or experience. In Ghana and other places in sub-Saharan Africa, this knowledge base is critical, given that health literacy knowledge was found to be very low among both nursing groups in this study. Future research can extend this study by examining how the various correlates of health literacy knowledge among student and practising nurses evolve in clinical and nursing education settings.

## Figures and Tables

**Table 1:** Descriptive statistics and differences between student and practising nurses as regards the variables included in the study by Chi-Square analyses ^#^.

Variable	Student Nurses *N* = 477	Practising Nurses (*N* = 399)	*p*-Value	Overall (*N* = 876)
	*N* (%)	*N* (%)		*N* (%)
**Age (in years)**			**0.001** ^**a**^	
*Mean (SD)*	*23.3 (3.4)*	*29.0 (5.1)*		*26.2 (4.3)*
*Minimum–Maximum values*	*18–46*	*21–59*		
**Sex**			**0.035**	
Male	224 (47.0)	156 (39.3)		380 (43.5)
Female	253 (53.0)	241 (60.7)		494 (56.5)
**Region of work/school**			**0.008**	
Ashanti Region	78 (16.4)	88 (22.3)		166 (19.1)
Central Region	10 (2.1)	8 (2.0)		18 (2.1)
Eastern Region	12 (2.5)	13 (3.3)		25 (2.9)
Greater Accra Region	207 (43.5)	97 (24.6)		304(34.9)
Northern Region	61 (12.8)	132 (33.4)		193 (22.2)
Upper East	38 (8.0)	13 (3.3)		51 (5.9)
Upper West	2 (0.4)	2 (0.5)		4 (0.5)
Brong Ahafo Region	24 (5.0)	27 (6.8)		51 (5.9)
Western Region	6 (1.3)	7 (1.8)		13 (1.5)
Volta Region	38 (8.0)	8 (2.0)		46 (5.3)
**Area of residence**			0.168	
Urban	348 (73.0)	308 (77.2)		656 (74.9)
Rural	129 (27.0)	91 (22.8)		220 (25.1)
**Educational attainment**			**0.005**	
Certificate in nursing	84 (17.6)	104 (26.0)		188 (21.5)
Diploma in nursing	311 (65.2)	211 (53.1)		522 (59.6)
Bachelor’s degree	74 (15.5)	76 (18.9)		150 (17.1)
Master’s degree	8 (1.7)	8 (2.1)		16 (1.8)
**Marital status**			**0.001** ^**L**^	
Married	32 (6.7)	151 (37.8)		173 (19.7)
Divorced	10 (2.1)	6 (1.5)		16 (1.8)
Windowed	1 (0.2)	2 (0.6)		4 (0.4)
Separated	11 (2.3)	14 (3.5)		25 (2.8)
Living together as married	5 (1.1)	10 (2.4)		14 (1.6)
**Monthly Income/stipend** (if employed) GH¢			**0.001** ^**a**^	
*Mean (SD)*	*392.28 (81.38)*	*1150 (705.94)*		
*Minimum–Maximum values*	*0–740*	*0–4000*		
**Religiosity**			**0.001**	
Strongly agree	114 (23.9)	127 (31.9)		238 (27.2)
agree	200 (42.1)	186 (46.6)		385 (44.0)
Neither agree nor disagree	80 (16.8)	46 (11.5)		128 (14.6)
Disagree	48 (10.1)	20 (5.0)		70 (8.0)
Strongly disagree	20 (4.2)	20 (5.0)		55 (6.3)
**Cultural values**			0.995	
Strongly agree	45 (9.5)	17 (4.2)		85 (9.7)
agree	237 (49.6)	201 (50.3)		438 (50.0)
Neither agree nor disagree	83 (17.3)	69 (17.2)		151 (17.2)
Disagree	72 (15.0)	57 (14.2)		128 (14.6)
Strongly disagree	41 (8.6)	33 (8.3)		74 (8.5)
**Trust in others**			**0.001**	
Yes	198 (41.5)	103 (25.7)		301 (34.4)
No	279 (58.5)	296 (74.3)		575 (65.6)
**Membership of an association**			0.251	
Yes	321 (67.3)	253 (63.4)		574 (65.5)
No	156 (32.7)	146 (36.6)		302 (34.5)
**Socioeconomic Status (SES)**			**0.005** ^**a**^	
Mean (SD)	*5.3 (2.0)*	*5.7 (1.7)*		*5.5(1.9)*
*Minimum–Maximum values*	*0–10*	*0–10*		*0–10*
**Nursing speciality**			**0.001**	
General Nursing	265 (55.9)	184 (46.2)		449 (51.4)
Midwifery	71 (14.5)	48 (12.1)		119 (13.6)
Mental Health Nursing	49 (10.3)	125 (31.4)		174 (19.9)
Community Nursing	41 (8.6)	28 (7.0)		69 (7.9)
Public Health Nurse	28 (5.9)	9 (2.3)		37 (4.2)
Occupational therapy	19 (4.0)	--		19 (2.2)
Clinical nurse assistant	2 (0.4)	2 (0.5)		4 (0.5)
Ophthalmology	--	2 (0.5)		2 (0.2)
**Years of experience ^**			--	
*Mean (SD)*	*--*	*4.8 (3.8)*		*4.8 (3.8)*
*Minimum–Maximum values*	*--*	*1–27*		*1–27*
**Year of study ^^**			--	
2	219 (46.4)	--		219 (46.4)
3	227 (48.1)	--		227 (48.1)
4	26 (5.5)	--		26 (5.5)
**Health status**			0.790	
Poor	33 (6.9)	22 (5.5)		55 (6.3)
Fair	96 (20.1)	87 (21.8)		183 (20.9)
Good	204 (42.8)	157 (39.3)		361 (41.2)
Very good	100 (21.0)	103 (25.8)		203 (23.2)
Excellent	44 (9.2)	30 (7.5)		74 (8.4)
**Physical activities**			**0.024**	
Never	96 (20.1)	97 (24.5)		193 (22.1)
Once	139 (29.1)	112 (28.3)		251 (28.8)
Several times in the month	81 (17.0)	101 (25.5)		182 (20.8)
Several times a week	104 (21.8)	41 (10.4)		145 (16.6)
Daily	57 (11.9)	45 (11.4)		102 (11.7)
**Use of curative healthcare**			**0.001**	
Never	85 (17.9)	77 (19.3)		162 (18.5)
Not often	232 (48.8)	271 (67.9)		496 (56.8)
Often	128 (26.9)	40 (10.0)		174 (19.9)
Countless times	30 (6.3)	11 (2.8)		42 (4.8)
**Health system satisfaction**			**0.001**	
Completely dissatisfied	59 (12.9)	78 (19.5)		137 (16.0)
Very dissatisfied	95 (20.1)	82 (20.6)		177 (20.7)
Fairly dissatisfied	77 (16.8)	91 (22.8)		168 (19.6)
Neither satisfied nor dissatisfied	51 (11.1)	38 (9.5)		89 (10.4)
Fairly satisfied	138 (30.1)	91 (22.8)		229 (26.7)
Very satisfied	24 (5.2)	11 (2.7)		35(4.1)
Completely satisfied	14 (3.1)	8 (2.0)		22 (2.6)
**Heard of health literacy**			**0.038**	
Yes	372 (78.3)	285 (71.4)		659 (75.4)
No	103 (21.7)	114 (28.6)		215 (24.6)
**Health literacy knowledge (HLKES)**			**0.001** ^**a**^	
*Mean (SD)*	*5.8 (2.6)*	*7.4 (3.0)*		*6.6 (2.9)*
*Minimum –Maximum values*	*0–13*	*0–17*		*0–17*

^ Relevant to only practising nurses. ^^ Relevant to only student nurses. ^a^ Value is based on independent *t*-test; ^L^ value is based on Likelihood Ratio; ^#^ Some figures may not add up to the total due to missing data. Bold values denote statistical significance at *p* < 0.05.

**Table 2:** Factors associated with health literacy knowledge among student and practising nurses in Ghana by ordinal logistics regression analyses.

	Student Nurses ^				Practising Nurses				Overall Sample ^			
	B	95% CI	Stand. Error	Odds Ratio ^b^	B	95% CI	Stand. Error	Odds Ratio ^b^	B	95% CI	Stand. Error	Odds Ratio ^b^
**Personal and socio-cultural characteristics**												
Age	--	--	--	--	−0.007	−0.106, 0.091	0.050	0.993	0.055	−0.016, 0.127	0.037	1.057
Sex												
Male	−0.499 **	−0.876, −0.122	0.192	0.607	--	--	--	--	−0.394	−0.851, 0.062	0.233	0.674
Female (ref)												
Marital status ^^												
Married	--	--	--	--	−0.148	−2.006, 1.709	0.948	0.862	−1.930*	−3.780, −0.080	0.944	0.145
Divorced	--	--	--	--	−0.830	−4.091, 2.432	1.664	0.436	−1.563	−3.631, 0.505	1.055	0.210
Separated	--	--	--	--	−1.655	−4.032, 0.723	1.213	0.191	−1.050	−2.333, 0.233	0.655	0.350
Living together as married	--	--	--	--	−0.655	−2.530, 1.220	0.957	0.519	−1.357 *	−2.642, −0.071	0.656	0.257
Single (ref)												
Income	0.526	−0.153, 1.206	0.347	1.692	0.504	−0.699, 1.706	0.614	1.655	0.001 **	0.000, 0.001	<0.001	1.001
Religiosity	0.112	−0.043, 0.267	0.079	1.119	0.123	−0.167, 0.412	0.148	1.131	0.173	−0.054, 0.401	0.116	1.1889
Trust in others												
Yes	−0.874 ***	−1.265, −0.483	0.199	0.417	−1.252 **	−1.978, −0.526	0.370	0.286	−0.939 ***	−1.257, −0.621	0.162	0.391
Membership of an association												
Yes	−0.297	−0.704, 0.110	0.208	0.743	--	--	--		0.161	−0.315, 0.637	0.243	1.175
SES	--	--	--		--	--	--		−0.221 **	−0.361, −0.082	0.071	0.802
Cultural values	0.276 ***	0.113, 0.439	0.083	1.318	0.357**	0.065, 0.650	0.149	1.429	0.378 **	0.156, 0.601	0.114	1.459
Area of residency												
Rural	--	--	--		−0.025	−0.685, 0.636	0.337	0.975	--	--	--	
Urban (ref)												
**Nurse Characteristics**												
Practicing experience	--	--	--		0.612 **	0.386, 0.974	0.260	1.844	−0.045	−0.129, 0.038	0.043	0.956
Year of study	0.244 *	0.101, 0.568	0.094	1.276	--	--	--		--	--	--	--
Type of nurse												
Student nurse	--	--	--		--		--		−0.577 *	−0.912, −0.384	0.260	0.562
Practising nurse (Ref)												
Educational attainment												
Certificate in nursing	−0.461	−2.256, 1.334	0.916	0.631	--	--	--		--	--	--	--
Diploma in nursing	−0.155	−1.914, 1.604	0.898	0.856	--	--	--		--	--	--	--
Bachelor’s degree	0.030	−1.785, 1.844	0.926	1.030	--	--	--		--	--	--	--
Masters (ref)												
**Health and health system**												
Health status	--	--	--		0.158	−0.180, 0.496	0.173	1.171	0.147	−0.086, 0.381	0.119	1.158
Use of curative care use	−0.236 *	−0.457, −0.015	0.113	0.790	--		--		−0.146	0.496, −0.205	0.179	0.864
Physical activities					0.175	−0.078, 0.428	0.129	1.191	--	--	--	
*Nagelkerke’s R^2^*	*0.263*				*0.245*				*0.275*			

Notes: * *p* < 0.05. ** *p* < 0.01. *** *p* < 0.001; ^ Controlled for the region of work/school; -- Variable excluded because of no initial correlation with health literacy knowledge; ^^ Widowed category was automatically excluded due to redundancy; *CI =* confidence interval; **^b^** Odds ratio computed using the resource provided by De Coster and Iselin [35].

## Data Availability

The data presented in this study are available on reasonable request to the corresponding author.
